# The Risks of Phosphate Enemas in Toddlers: A Life-Threatening Unawareness

**DOI:** 10.3390/children11030349

**Published:** 2024-03-15

**Authors:** Alessandro Zago, Alessandro Agostino Occhipinti, Matteo Bramuzzo, Viola Ceconi, Vincenzo Colacino, Egidio Barbi, Federico Poropat

**Affiliations:** 1 Department of Medicine, Surgery and Health Sciences, University of Trieste, 34149 Trieste, Italy; alessandroagostino.occhipinti@burlo.trieste.it (A.A.O.); viola.ceconi@burlo.trieste.it (V.C.); egidio.barbi@burlo.trieste.it (E.B.); 2 Department of Pediatrics, Institute for Maternal and Child Health—IRCCS “Burlo Garofolo”, 34137 Trieste, Italy; matteo.bramuzzo@burlo.trieste.it (M.B.); federico.poropat@burlo.trieste.it (F.P.); 3 Azienda Sanitaria Universitaria Friuli Centrale, 33100 Udine, Italy; vincenzo.colacino@asufc.sanita.fvg.it

**Keywords:** phosphate-containing enemas, constipation, side effects, pediatric gastroenterology

## Abstract

Background: While oral laxatives represent the first-line treatment of fecal impaction, enemas are frequently used in clinical practice in pediatric emergency departments (PEDs) and by family pediatricians (FPs). Objectives: Phosphate-containing enemas (PcEs) are commonly employed, even causing the risk of rare but lethal toxicity. We investigated pediatricians’ awareness of PcE risks. Methods: We conducted an online survey by sending a multiple-choice questionnaire to the referents of 51 PEDs and 101 FPs. We collected and compared the answers with recommendations reported by the Italian Drug Agency (AIFA) and the available literature about PcE administration. Results: Of the institutions and pediatricians receiving the questionnaire, 23 PEDs (45%) and 63 FP (62.3%) participated in the survey. Of PEDs, 95% and 33.0% of FPs treated fecal impaction with PcE. Moreover, 54% of PEDs and 86.0% of FPs did not provide treatment according to the AIFA recommendations for the daily dose. Conclusions: This study shows limited pediatricians’ awareness of the potential risks related to PcE.

## 1. Introduction

Constipation is a frequent disorder in children, with a prevalence of up to 29% in healthy kids, and 60% in children with cerebral palsy and severe cognitive impairment [1]. Regarding pediatric patients, 95% of cases are represented by functional constipation, a clinical sign consequent to a behavioral dysfunction rather than the presence of an organic systemic or gastroenterological condition.

Constipation is defined by the Rome IV criteria as a difficult, infrequent, and painful passage of stools in the bowel tract that can have a negative impact on children’s quality of life [2,3]. Children may have a retentive/withholding behavior triggered by fear of evacuation due to anal fissuration, hard stools, or pain perceived in the anorectal area during toileting, or secondary to the presence of negative or unfamiliar stimuli in bathrooms at schools or outside familiar environments.

Therefore, the therapeutic pathway for treating constipation is represented by disimpaction, behavioral education, and maintenance therapy. Constipation is associated with a compromised quality of life, detected by the administration of validated questionnaires due to several mechanisms, such as the stigmatizing consequences of fecal incontinence, a typical and frequent sign of constipation secondary to soft fecal overflow around the impacted stool, and the chronicity of the condition [4].

The role of caregivers is well known, as evidence shows that changes in parental attitude toward their children’s problem of constipation can have a marked positive impact on the symptoms and treatment outcomes [5]. Fecal incontinence, the involuntary leak of a small quantity of stools also known as “soiling”, defined as retentive in the context of constipation, is indeed an underestimated and misinterpreted sign [6]. In fact, caregivers may think that their children are uncooperative or even have chronic diarrhea; the consequence is diagnostic retardation, which negatively impacts on the management [7].

Regarding children with disability, constipation constitutes the main cause of pain in 10% of children with cerebral palsy [8], who are more likely to have treatment-resistant constipation [9]. The condition could be secondary to immobilization, spasticity, and the use of drugs that reduce intestinal motility, such as myorelaxants, anticholinergics used for sialorrhea, or opioids for pain [10].

Fecal impaction can be defined as a large amount of stool that can be diagnosed clinically, and involves 30 to 75% of children with chronic constipation [11]. Fecal impaction is a common cause of seeking emergency medical advice. Even if abdominal pain does not belong to the diagnostic criteria for FC, it is the most frequent complaint [12], to the extent that studies show that nearly a quarter of children who access care at PEDs for abdominal pain have functional constipation [13]. The clinical presentation can mimic common surgical conditions like appendicitis, or more rarely inflammatory bowel disorders, and FC represents a differential diagnosis of surgical or other organic conditions that can lead to severe morbidity. For this reason, at any age, it is mandatory to consider red flags and alarm signs suggestive of an organic condition. Medical history and a physical exam should include and evaluate the lumbosacral, perianal, and abdominal regions, and a high index of suspect for signs of spinal dysraphism, abdominal masses, malignant infiltration of the vertebrae or the spinal cord, infections such as spondylodiscitis, or the onset of a Guillain Barré syndrome should be considered [14,15,16,17,18]. As a rule, digital rectal examination should be avoided because it could be distressing for the child, except for the diagnosis of Hirschsprung’s disease where explosive stools, abdominal distension, and a history of recurrent enterocolitis are highly suggestive of the condition [19]. Moreover, refractory constipation should be considered a red flag for organic conditions.

After establishing the etiology, disimpaction and maintenance therapy are the subsequent steps. First-line treatment for fecal disimpaction is represented by oral laxatives [20]; however, enemas could be a practical, easy, and inexpensive diagnostic and therapeutic tool to treat constipation and relieve pain promptly. Moreover, enemas are also employed during bowel preparation preceding gastrointestinal surgery or endoscopy. According to the ESPGHAN guidelines, 1 to 1.5 g/kg/day polyethylene glycol (PEG) orally for 3–6 days is recommended, and at least a one-week therapy is suggested. As a rule, the use of oral PEG is preferred over enemas, being more tolerated by children, and enemas are not routinely used in several countries. PEG is a high-molecular-weight and non-absorbable polymer; each molecule can create a hydrogen bond with 100 molecules of water contained in the intestinal lumen, making stools more hydrated. As a matter of fact, an increase of 10% in stool hydration can make them softer and simpler to evacuate significantly, an effect that improves the passage of stools without affecting electrolyte metabolism [21]. The onset of PEG action is not immediate, so it is important to educate caregivers that the molecule reaches its effect after 24–48 h to achieve adequate compliance [22].

Moreover, this interval could be useful for educating the child about toilet training measures, combining education and maintenance therapy. Indeed, behavioral and psychological factors have a strong role in constipation, which could constitute the epiphenomenon of a mental health issue, a psychological maladjustment, or a sign of abuse [23,24]. In a study involving a cohort of patients aged between 8 and 12 years, “self-efficacy”, which consists of confidence in having resources to solve a problem or manage a difficult situation, was a predictor of treatment outcomes, as children empowered by the pediatric gastroenterologist specialist during the visits were more aware of the causes and solutions to cope with constipation [25]. Remarkably, an early and effective disimpaction therapy correlates with a better outcome in the long term [26]. Even if guidelines identify oral laxatives and enemas as equally effective, oral medications are preferred because they are less invasive and uncomfortable for children. On the other hand, the use of PcE enables clinicians to achieve a fast and prompt resolution of symptoms [13]. While phosphate-containing enemas are not used in several countries, their use as a therapeutic tool is widely described and reported in the ESPGHAN guidelines. As a matter of fact, enemas are associated with an increased rate of revisits to PEDs [27] and less resolution of constipation in the long term. This could be due to the topical action limited to the rectum and children’s reluctance to receive such invasive therapy for a long time. Hence, the benefit of phosphate expires just after 24 h [22]. In addition, PcE causes less frequently fecal incontinence if compared to oral medications; the administration of PEG, especially in the absence of an adequate prior disimpaction, can result in paradoxical diarrhea [11].

The selection of the enema type is based more on physicians’ preferences and local practice patterns than proven differences in efficacy or safety [28].

**Figure 1:**
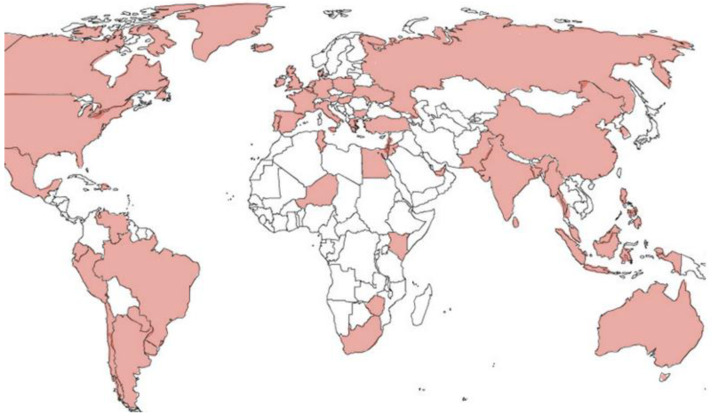
Nations in which the use of phosphate enema is allowed.

Among the different options, phosphate-containing enemas (PcEs), commercialized worldwide with different names, are often used in clinical practice, also being available over the counter (Figure 1, Table A1, see Appendix A).

PcEs act by triggering peristalsis and allowing for defecation, usually in a few minutes, through an osmotic mechanism. Indeed, inorganic phosphate reduces the osmolarity gap and enables the shift of the fluid from systemic circulation to the intestinal lumen. While not always foolproof, the quick onset of defecation should allow for the gut to be preserved from prolonged exposure to high phosphate doses. However, intestinal phosphate absorption may occur, with the development of hyperphosphatemia and severe hypocalcemia.

A systematic review of the use of PcE reports 12 deaths, along with other patients needing intensive care assistance due to severe hypotension, acute kidney impairment or coma [29]. Adverse effects are reported to be related to several specific risk factors, such as ages under 5 years, chronic renal diseases, and alterations in intestinal motility. Moreover, accidental overdoses have also been reported [30,31,32,33,34].

Among the different regulation agencies, the Italian Drug Agency (AIFA) and the Food and Drug Administration warned against the use of PcE in children under 3 and 2 years, respectively, with a recommended dose of 30–60 mL in children younger than 12 years and 120 mL in adolescents once a day [35,36].

At our institute, we routinely use phosphate-containing enemas in the context of PEDs and before surgical procedures. Prompted by a recent critical event of a toddler experiencing life-threatening hypocalcemia after being treated with two 120 mL phosphate enemas at another hospital, we realized that many of our physicians were not fully aware of these risks. While the clinical indications and adverse effects of PcE are well described, to the extent that there are clear limitations in their use, less is known about the awareness of the possible life-threatening effects. Considering the fact that those medications are available over the counter, informing and educating caregivers is an important hint for both PED and family pediatricians. Moreover, this study could be of interest.

For this reason, we performed an online investigation addressing the issue of awareness of phosphate-related risks in a network of Italian PEDs and in a sample of family pediatricians (FPs).

## 2. Materials and Methods

### 2.1. Study Design

We conducted a multi-center electronic survey to evaluate the frequency of the use of PcEs across Italian PEDs and among an arbitrary sample of Italian FPs from January to June 2023.

### 2.2. Setting and Population

The study population included pediatricians working at different PEDs and family pediatricians. Residents were excluded from the survey.

### 2.3. Data Collection Procedures

**Figure 2:**
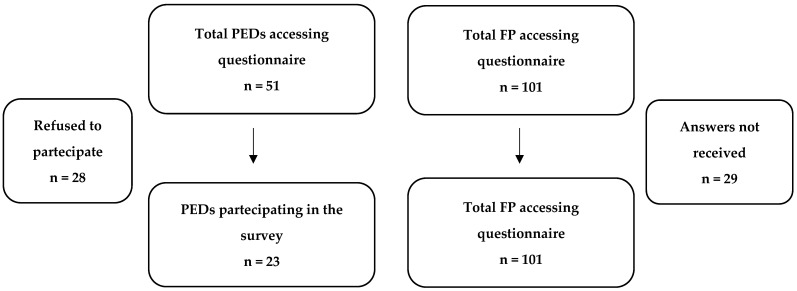
Diagram showing the study design.

Pediatricians were asked to complete a multiple-choice questionnaire collected using “Google Forms” by sharing an online link via e-mail. We contacted 51 PEDs (Figure 2) through an institutional referent and 101 FPs. A single referent answered on behalf of his/her PED protocols or practices, and each FP answered about his/her practice.

All participants submitted the questionnaire anonymously or without declaring the name of the PED. We collected all the interviewers’ answers and compared them with the recommendations reported by the AIFA and the available literature about PcE administration.

### 2.4. Data Analysis

The questionnaire was made up of 6 multiple-choice questions regarding the epidemiology and frequency of the use of PcE in clinical practice, asking how many pediatricians used enemas and PcE specifically in their clinical practice, the awareness of their use in terms of minimum age of administration, weight and age, maximum daily dose and adverse reactions.

The two questionnaires were different as FPs were not required to answer to the last question, i.e., “Have you ever experienced substantial adverse reactions using phosphate-containing enemas, like drowsiness or hypocalcemia?”, since we hypothesized that they lacked the tools to properly diagnose hypocalcemia and that children with severe side effects would have been admitted to a PED.

### 2.5. Ethics

Approval for data collection was obtained by the Ethical Committee of IRCCS Burlo Garofolo (ECC 49/2022, seduta IRB 02/2022, D.D. 16 March 2022).

## 3. Results

Of the 51 Italian PEDs, 23 (45%) and 63 of 101 (62.3%) FPs participated in this survey by answering the multiple-choice questionnaire.

The questionnaire and the answers are shown in Table 1.

## 4. Discussion

This study shows that while PcEs are primarily used at hospitals and family practice clinics, many pediatricians are unaware of their potential risks.

### 4.1. Use of PcEs within the Clinical Practice

The first finding was that 95% of institutions employed an enema for the disimpaction of children with acute abdominal pain in a critical setting, rather than oral laxative treatment. This approach appears reasonable since it provides an immediate relief of symptoms and helps rule out other possible causes of acute abdominal pain at the ED. In this context, PcEs are used in 50% of the interviewed centers.

Remarkably, more than half of the institutions did not formally recommend any limitation in their use, thus ignoring the minimal age cut-off defined by the AIFA. Indeed, 36% of the centers did not formally consider 2 years of age as the minimum cut-off and 10% did not assume any age as a limit for administration.

Similarly, 46.5% of the centers did not report a protocol to administer a dose per kilo as proposed by the ESPGHAN which suggested not exceeding the 2.5 mL/kg dose.

In family care setting, the use of enemas was scarce, with two-thirds of the respondents not using PcEs. Even so, according to this survey, 23% of the FPs using PcEs would still not limit their use under three years of age. From this perspective, it should be considered that the use of PcE, and rectal medications in general, could be an unusual practice for several pediatricians around the world.

### 4.2. Pathogenesis of Damage Related to Phosphate Enemas

In the past, it had been erroneously postulated that phosphate contained in an enema was poorly absorbed, underestimating the fact that retention times may count even more than the absolute dose [37,38].

Phosphate is an intracellular anion, mainly stored within the mineral hydroxyapatite in the bone. There are several causes of hyperphosphatemia due to increased phosphate load, such as enema administration or tumor lysis syndrome, reduced kidney excretion, or a transcellular shift. The main effects of acute hyperphosphatemia from an excessive external load are represented by its effects on calcium metabolism. Calcium homeostasis is regulated by two systems: the renal system, through tubular reabsorption; and the intestinal system, with an active transport system. Predisposing conditions, such as chronic kidney disease, can impair kidney function and, consequently, phosphate elimination. The volume loss and high phosphate intake could be responsible for hypertonic dehydration. In the same way, changes in the metabolism of other ions, such as calcium and magnesium, with the development of hypocalcemia as phosphate forms complexes with the serum calcium, is responsible for systemic symptoms such as spasms, tetany, seizures, and arrhythmia. The high level of phosphate concentration, with 63.3 mg/dL being the highest reported concentration [39], higher than the levels observed in chronic conditions causing hyperphosphatemia, is pathognomonic of acute administration or intoxication.

In most cases, hyperphosphatemia is transient, with a fall in the normal range for most patients within a few hours, while the concentration of serum calcium, potassium, and creatinine fluctuates but generally remains within the normal range. Remarkably, these effects are more common and potentially more severe in infants, and patients with gastrointestinal motility disorders, renal or neurological impairment, determining an unpredictable rise in hematic phosphate and a consequent fall in calcemia [29,40]. However, severe and fatal reactions have also been reported with small doses in infants without predisposing gastrointestinal or renal diseases [41,42].

### 4.3. Clinical Manifestations

Clinical features of intoxication are often initially nonspecific. Two-thirds of symptomatic children have a decreased level of consciousness a few minutes after enema administration. At the same time, tetany or other symptoms of hypocalcemia and hypotension have been reported in almost half and one-third of the patients, respectively. Kidney injuries, requiring dialysis, QT prolongation, cardiac arrest, and death, have also been described in a limited number of pediatric cases [41]. It may also be speculated that the reported cases do not necessarily represent the whole spectrum of adverse events and that more cases may have been unreported.

### 4.4. Limitations of the Study

This investigation has some limitations. The response rate of both PEDs and FPs was about 50%, representing only a partial and not homogeneous sample of what is performed in clinical practice; nonetheless, third-level pediatric hospitals of the country are included in the sample. Moreover, the use of phosphate-containing enemas, and rectal medications in general, could be an unusual practice for several pediatricians around the world. In addition, the answers regarding PEDs were provided by a doctor only referring to each institution’s policies and protocols, and may only reflect some physicians’ approaches partially. In this regard, some PED centers did not answer all the questions. Furthermore, this being a merely descriptive study, no statistical analysis was performed. In addition, the level of awareness of Italian pediatricians may not correspond to that of colleagues from other countries. Eventually, since there are no similar studies regarding the awareness of PcE use among pediatricians, we were not able to calculate an “a priori” sample size. For these reasons, the results have a limited generalizability and should be interpreted considering the sample size and the different everyday practices.

## 5. Conclusions

While the potential side effects of PcEs are well described, this is the first study to analyze pediatricians’ awareness regarding the use of these tools.

This report suggests a lack of adequate awareness of the potentially life-threatening issue. This is even more relevant if we consider that PcEs are not a lifesaving treatment, with various safer alternatives available, both in terms of different enemas and oral laxatives.

Finally, the over-the-counter availability may expose inadequately informed parents to the risk of administering this treatment to their children.

In conclusion, while the use of PCEs in Italy within the PED setting is widespread, there is evidence of a lack of protocols limiting the correct dosage for age and weight, with limited knowledge also among a sample of FPs. This can expose children, especially toddlers, to serious adverse events. Educational and regulatory efforts should be made to improve the use of PcEs in children.

## Figures and Tables

**Table 1:** Questionnaire and the answers of the participants.

		PED			FP				
Question	Answer	%	Number of Centers	Cumulative %	Total	%	Number	Total	Cumulative %
Do you perform enemas in children with abdominal pain and suspicion of functional constipation?	yes	95	22		23	36	23	63	
	no	5	1			64	40		
Do you use phosphate-containing enemas?	yes	50	11		22	32	20	63	32
	no	50	11			68	43		
Is there a minimum age under which phosphate-containing enemas should not be used?	1 yo	36	4	64	11	24	15	63	49
	2 yo	36	4			29	20		
	3 yo	18	2			23	14		
	no	10	1			11	7		
	I don’t know					11	7		
Is the dose of phosphate-containing enemas weight-dependent or age-dependent?	yes	54	6	46	11				
	no	46	5						
Does a maximum daily dose exist for phosphate-containing enemas?	1	45	5	55	11	14	9	63	54
	2	27	3			23	15		
	3	10	1			20	12		
	no	18	2			24	15		
	I don’t know					19	12		
Have you ever experienced substantial adverse reactions using phosphate-containing enemas, like drowsiness or hypocalcemia?	yes								
	no	100							

## Data Availability

The data presented in this study are available on request from the corresponding author. The data are not publicly available due to privacy reasons.

## Appendix A

**Table A1:** Countries in which phosphate enemas are available over the counter.

Country	Name
United Arab Emirates	Fleet Saline Enema Relief
Argentina	Novonil Prontonema
Austria	Clysmol
Australia	Travad
Bangladesh	Anema
Belgium	Cleen Colowash Fleet
Bulgaria	Enema Balkan
Brazil	Enema jp Enemaplex Enemed Fleet L-enema Phosphoenema
Canada	Fleet Enema
Chile	Casen Enema Adultos Fleet Forflow Phosfoenema
China	Dibasic sodium phosphate Evac Fleet Sodium phosphates rectal
Colombia	Enematrol Forflow Travad
Denmark	Cleenema
Dominican Republic	Enema Fleet
Ecuador	Fleet
France	Normacol lavement enfants
United Kingdom	Sodium phosphate
Germany	Klistier Fresenius Kabi Klysma
Greece	Bioklism Klysmol
Hong Kong	Fleet
Indonesia	Fosen Purgatix
Ireland	Cleenema ready to use Fleet
Israel	Fleet
India	Practo Clyss Royal
Italy	Clisflex Clisma fleet
Jordan	Fletchers Phosphat Klysmol Phosphate
Kenya	Enemax
Republic of Korea	Fleet
Lebanon	Alfa Clyss Fleet PractoClyss
Luxembourg	Cleen Fleet
Mexico	Fleet fosf-sodio
Malaysia	Fleet
Nigeria	Enemax Enemax(p)
The Netherlands	Colex enema Fleet
Peru	Adulax Lainema Laxoven Laxoven Pediatrico Movilax
Philippines	Fleet
Pakistan	Fast Rapida
Poland	Fleet PROCTANAL
Portugal	Enema fleet Fleet
Paraguay	Fleet enemas adultos Fleet enemas ninos Fosfocol
Russia	Enema clean
Spain	Enema Casen Infantil Lainema
Singapore	Fleet
Thailand	Fleet
Tunisia	Normacol lavament
Turkey	B.T. Enema Fleet
Taiwan	Evac Fleet
Ukraine	Normacol
Uruguay	Fosfocol
USA	Fleet Enema Extra Fleet Enema Fleet Pedia-Lax Enema GoodSense Enema LaCrosse Complete
Bolivarian Republic of Venezuela	Clismalax Fleet Fleet enema Fosfolit
South Africa	Lenolax Lenolax Paediatric
Zimbabwe	Fosenema
